# Species‐specific plant‐mediated effects between herbivores converge at high damage intensity

**DOI:** 10.1002/ecy.3647

**Published:** 2022-03-21

**Authors:** Jinlong Wan, Jiahui Yi, Zhibin Tao, Zhikun Ren, Evans O. Otieno, Baoliang Tian, Jianqing Ding, Evan Siemann, Matthias Erb, Wei Huang

**Affiliations:** ^1^ CAS Key Laboratory of Aquatic Botany and Watershed Ecology, Wuhan Botanical Garden Chinese Academy of Sciences Wuhan China; ^2^ Center of Conservation Biology, Core Botanical Gardens Chinese Academy of Sciences Wuhan China; ^3^ University of Chinese Academy of Sciences Beijing China; ^4^ School of Life Sciences Henan University Kaifeng China; ^5^ Department of Biosciences Rice University Houston Texas USA; ^6^ Institute of Plant Sciences, University of Bern Bern Switzerland

**Keywords:** above‐ and belowground herbivore interaction, conspecific and heterospecific herbivore interaction, density‐dependent effect, herbivore‐induced specific response, identity and density of herbivores, plant‐induced defense, plant‐herbivore interaction, resistance and susceptibility

## Abstract

Plants are often exposed to multiple herbivores and densities of these attackers (or corresponding damage intensities) often fluctuate greatly in the field. Plant‐mediated interactions vary among herbivore species and with changing feeding intensity, but little is known about how herbivore identity and density interact to determine plant responses and herbivore fitness. Here, we investigated this question using *Triadica sebifera* (tallow) and two common and abundant specialist insect herbivores, *Bikasha collaris* (flea beetle) and *Heterapoderopsis bicallosicollis* (weevil). By manipulating densities of leaf‐feeding adults of these two herbivore species, we tested how variations in the intensity of leaf damage caused by flea beetle or weevil adults affected the performance of root‐feeding flea beetle larvae and evaluated the potential of induced tallow root traits to predict flea beetle larval performance. We found that weevil adults consistently decreased the survival of flea beetle larvae with increasing leaf damage intensities. In contrast, conspecific flea beetle adults increased their larval survival at low damage then decreased larval survival at high damage, resulting in a unimodal pattern. Chemical analyses showed that increasing leaf damage from weevil adults linearly decreased root carbohydrates and increased root tannin, whereas flea beetle adults had opposite effects as weevil adults at low damage and similar effects as them at high damage. Furthermore, across all feeding treatments, flea beetle larval survival correlated positively with concentrations of carbohydrates and negatively with concentration of tannin, suggesting that root primary and secondary metabolism might underlie the observed effects on flea beetle larvae. Our study demonstrates that herbivore identity and density interact to determine systemic plant responses and plant‐mediated effects on herbivores. In particular, effects are species‐specific at low densities, but converge at high densities. These findings emphasize the importance of considering herbivore identity and density simultaneously when investigating factors driving plant‐mediated interactions between herbivores, which advances our understanding of the structure and composition of herbivore communities and terrestrial food webs.

## INTRODUCTION

Upon herbivore attack, plants undergo substantial changes in primary and secondary metabolites (Johnson et al., 2016; Karban & Myers, 1989; Schwachtje & Baldwin, 2008). These induced responses can affect the performance of conspecific and heterospecific herbivores in both above‐ and belowground compartments and thereby drive herbivore community dynamics (Poelman & Dicke, 2014; Poelman & Kessler, 2016; Van Zandt & Agrawal, 2004). However, plant‐mediated interactions between herbivores remain difficult to predict, because the factors that determine these outcomes are not fully understood (Biere & Goverse, 2016; Haukioja, 1991; Nykänen & Koricheva, 2004).

A large body of evidence documents that herbivores elicit herbivore‐species‐specific plant responses (Agrawal, 2000; Ali & Agrawal, 2012; Karssemeijer et al., 2020; Kessler & Halitschke, 2007). Thus, herbivore identity is considered a key determinant of plant‐mediated interactions between herbivores (Kafle et al., 2017; Stout et al., 1997; Viswanathan et al., 2007). Some herbivores increase toxins and anti‐digestive compounds or decrease nutrients causing induced resistance to herbivores that arrive later (Denno et al., 2000; Soler et al., 2005), while others have opposite effects on these metabolites resulting in induced susceptibility to later attack (Robert et al., 2012a; Su et al., 2018). Such opposing effects are partially caused by highly specific herbivore‐associated cues (e.g., saliva, frass, and odor as well as feeding behavior and damage pattern) that are released and exert control during feeding (Basu et al., 2018; Erb et al., 2012a; Soler et al., 2013). Some of these cues are recognized specifically by plants and result in the elicitation of defense responses, while others can suppress plant defenses (Erb & Reymond, 2019; Kant et al., 2015).

A second factor that shapes plant‐mediated effects is herbivore density (Kaplan et al., 2008a; Masters, 1995). The initial herbivore‐species‐specific responses of plants are commonly assumed to be more intense at higher densities as specific herbivore‐derived cues are stronger (e.g., saliva; Karban & Baldwin, 1997, Agrawal & Karban, 2000, Eisenring et al., 2017). At the same time, high herbivore densities can result in resource overexploitation, thus reducing the fitness of later arrivers through a simple lack of food (Johnson & Amarasekare, 2015; Kaplan & Denno, 2007; Ohgushi & Sawada, 1998). Resource overexploitation can affect herbivores feeding on the same tissues, but also herbivores feeding on different tissues such as leaves versus roots. For instance, aboveground herbivory often strongly decreases plant root growth with increasing feeding intensity (Huang et al., 2012a; Hunt‐Joshi & Blossey, 2005; Masters, 1995; Wilson et al., 2021). The net effect of herbivore density is therefore likely a combination of increasing plant responses to the herbivore and increasing resource overexploitation (Robert et al., 2012b).

Following the concept that density‐dependent effects of herbivores partially reflect species‐specific plant induction patterns, we would expect that density‐dependent effects are at least partially species‐specific. For herbivores that trigger induced resistance, one would expect that with higher density, induced resistance and thus negative effects on other herbivores would increase, and that this pattern would be further accentuated by resource overexploitation. For herbivores that trigger induced susceptibility, one would expect a positive effect on other herbivores at lower densities when the remaining food for subsequent arrivers would be enough, and that this pattern would be attenuated, counteracted, or even reversed at higher densities due to increasing resource overexploitation. To date, plant‐mediated interactions between herbivores have predominantly been investigated either for one attacking herbivore species under single or multiple densities or for many attacking species under a given density (Huang et al., 2014; Kaplan et al., 2007; McKenzie et al., 2013; Soler et al., 2005). To the best of our knowledge, however, species‐specific density effects on plant‐mediated interactions between herbivores have not been investigated, thus limiting our understanding and prediction of this important aspect of plant‐herbivore community dynamics.

Here, we examined the species‐specific impacts of herbivore density using *Triadica sebifera* (Euphorbiaceae, tallow tree, hereafter “tallow”) and its associated herbivores *Bikasha collaris* (Coleoptera: Chrysomelidae, hereafter “flea beetle”) and *Heterapoderopsis bicallosicollis* (Coleoptera: Attelabidae, hereafter “weevil”). In our previous work, we found that flea beetle adults feeding on leaves improved root quality (reducing resistance and increasing nutrients) and facilitated conspecific larvae feeding on the roots (Huang et al., 2012b, 2013; Sun et al., 2019). In contrast, weevil adults feeding on leaves decreased root quality and inhibited flea beetle larvae feeding on the roots (Huang et al., 2014). We thus selected these two herbivore species in this study and performed a growth chamber experiment within which we manipulated the density of weevil adults and flea beetle adults to cause varying feeding intensities. By evaluating the performance of flea beetle larvae on these damaged plants, we tested whether the plant‐mediated effects of adult weevil and flea beetle attack on subsequent arriving flea beetle larvae depend on the densities of these two adult herbivores. Furthermore, by analyzing the changes in root quality (primary and secondary metabolites) and quantity (biomass), we examined whether these traits mediated observed indirect effects on flea beetle larvae.

## MATERIAL AND METHODS

### Study system

Tallow is a rapidly growing, subtropical tree, which is native to east and southeast Asia. It is widely distributed in central and southern China and is especially abundant in natural areas (e.g., forest) and disturbed habitats (e.g., agricultural fields, roadsides, gardens). At the field survey site, Hubei located in central China (31.58° N, 114.18° E), tallow is attacked by multiple herbivores (Appendix S1: Table S1) and feeding intensity on leaves varies drastically, ranging from 0% to 71.5% (Appendix S1: Figure S1a). Flea beetle and weevil adults were two of the most abundant herbivores (Appendix S1: Figure S1b) and the abundance of these two herbivores were strongly positively correlated with leaf damaged area (Appendix S1: Tables S1 and S2, Figure S1c,d). More details for the methods, statistical analyses and results about the field survey are available in Appendix S1. Previous host range tests indicated that the flea beetle and weevil are monophagous specialists that exclusively feed on tallow (Huang et al., 2011; Wang et al., 2009).

Flea beetle adults feed on leaves causing small holes and oviposit on the soil surface at the base of tallow, and larvae feed on roots forming elongate tunnels (Huang et al., 2011). In Hubei province in central China, there are typically five generations per year with adults present beginning in late April until November when they start to overwinter in the rhizosphere (Huang et al., 2011). Under laboratory conditions, flea beetle adults can live more than 200 days on tallow and begin to oviposit about 17 days after eclosion (Wheeler et al., 2017). Adults oviposit in clutches every 3 days with about 24 eggs per clutch (Wheeler et al., 2017). The duration of the life cycle is about 33–36 days from egg to adult (egg, 8–9 days; larva, 17–18 days; pupa, 8–9 days; Huang et al., 2011).

Weevil adults feed on leaves making large scars. Female adults roll leaves for oviposition, and eggs, larvae and pupae develop inside the rolled leaves (Wang et al., 2009). In Hubei, there are six or seven generations per year with adults present beginning in May until October when they start to overwinter in the litter around tallow (Wang et al., 2009). Under laboratory conditions, weevil adults can live more than 80 days on tallow and begin to oviposit about 17 days after eclosion (Steininger et al., 2013). Adults oviposit 1–4 eggs per rolled leaf (Steininger et al., 2013). The duration of the life cycle is about 12–16 days from egg to adult (egg, 4–5 days; larva, 5–7 days; pupa, 3–4 days; Wang et al., 2009).

### Species‐specific impacts of herbivore density on herbivore interactions

We collected seeds from 20 trees at the field survey site that were separated by at least 50 m in November 2018. We randomly selected 100 seeds from each maternal tree, completely mixed them and soaked them in water with laundry detergent (10 g/L) for 2 days to remove waxy coats. Then, we placed them in moist sand at 4°C for 3 months to break dormancy (Huang et al., 2010). We sowed seeds in 50‐cell seeding trays filled with seedling substrate (Klasmann‐Deilmann GmbH, Geeste, Germany) in a growth chamber (14 h light/10 h dark with 24°/18°C temperature, relative humidity 50%–70%) at Wuhan Botanical Garden, Chinese Academy of Sciences, Hubei, China (30.61° N, 114.54° E). Four weeks later, we individually transplanted similar‐sized plants with four or five leaves into pots (14 cm in height, 12 cm in diameter) filled with a homogenized 50:50 mixture of seedling substrate and sand. Plants were watered every 2 days and were rearranged every week to eliminate possible impacts of environmental heterogeneity within the growth chamber.

We collected flea beetle adults and weevil rolled leaves at the field survey site in June 2019. Flea beetle adults were reared on 3‐month‐old tallow plants in a nylon mesh cage (1 × 1 × 1 m, 0.8 mm mesh sieve) in growth chamber with same conditions as already described. To obtain flea beetle larvae, naturally mated flea beetle adults were transferred to Petri dishes (10 cm in diameter, one pair per Petri dish). Each Petri dish contained a tallow leaf as food (Hubei population) and corrugated moist filter paper as oviposition substrate. We checked the leaves and oviposition substrates every 3 days and placed eggs into a new Petri dish with moist filter paper under constant darkness. Field collected flea beetle adults and laboratory reared newly hatched flea beetle larvae were used for the subsequent experiments. To ensure that we could obtain sufficient newly hatched flea beetle larvae simultaneously, we conducted egg collections on 300 pairs of mating adults. Weevil‐rolled leaves were put on wet hand towels enclosed within a nylon mesh cage (0.7 × 0.7 × 0.7 m, 0.8 mm mesh sieve). Newly emerged weevil adults were used for the subsequent experiments. To obtain sufficient newly hatched weevil adults, we collected more than 3000 rolled leaves in the field.

To examine herbivore identity and density dependent effects on plant induced resistance and susceptibility to flea beetle larvae, we conducted a bioassay by exposing flea beetle larvae to the roots of tallow seedlings that experienced prior leaf damage by different densities of weevil or flea beetle adults. Three weeks after transplantation, we selected plants with 10 fully expanded leaves, enclosed them individually in nylon mesh cages (14 cm in diameter, 40 cm in height, and 0.8 mm mesh sieve), and placed them in the growth chamber under the conditions described above. During the experiment, plants were watered as needed and were rearranged every week. Subsequently, we randomly assigned them to one of five densities of weevil adults, or one of five densities of flea beetle adults. Because per capita leaf removal is higher for weevils than flea beetles, we released 0, 2, 4, 6, or 8 newly emerged weevil adults or 0, 4, 10, 20, or 32 field collected flea beetle adults into each cage. There were 30 replicates for each combination (herbivory identity × herbivory density, 300 pots). Herbivores were allowed to feed on tallow for 1 week. To prevent oviposition by flea beetle adults into the soil, the mesh cage of each pot was sealed by string tied to the base of plant stem (below all leaves). We also sealed other pots to eliminate the possible impact of string.

One week after herbivore feeding, we removed all adults for both herbivores and assessed the leaf damaged area for each plant. The amount of damage was determined by visual estimate for each leaf by a 5% interval category, then averaging the visual estimates for all leaves per plant. Leaf damage at the highest density for both herbivores was around 70%, which was consistent with leaf damage levels observed in our field survey (Appendix S1: Figure S1a). Then, we punched a hole (1 cm in diameter, 2 cm in depth) in the soil at the base of each plant, transferred 10 newly hatched flea beetle larvae into the hole and covered them with moist soil. We recorded the number of emerging flea beetle adults and removed them every day. This process lasted 32 days, which was enough for flea beetle development from larva to adult.

### Species‐specific impacts of herbivore density on plant traits

To evaluate changes in root traits that influence flea beetle larvae, we measured root biomass and root primary and secondary metabolites of tallow seedlings that experienced prior leaf damage by different densities of weevil or flea beetle adults. We used the same procedures as described above with a different set of 300 plants (i.e., seedling transplantation, cage installation, adult herbivore addition and removal, leaf damage measurement). We cleaned roots with tap water then weighed, flash froze in liquid nitrogen, finely ground, and stored them at −80°C. Our previous studies indicated that primary metabolites and tannin play a critical role in mediating above‐ and belowground herbivore interactions on tallow (Huang et al., 2013, 2014). We determined glucose, fructose, and starch using their corresponding assay kits (Beijing Solarbio Science & Technology, Beijing, China). Protein was extracted by a plant protein extraction kit (Beijing Solarbio Science & Technology) and quantified by a protein assay kit (Takara Biomedical Technology, Beijing, China). All procedures followed the manufacturers' instructions. Tannin was measured using a radial diffusion assay (Hagerman, 1987) with a tannic acid standard (Sigma‐Aldrich, St. Louis, Missouri, USA). All chemical concentrations were expressed as mg/g fresh mass.

### Statistical analyses

To examine the species‐specific impacts of herbivore density on larval flea beetle performance (larval survival) and plant root traits (biomass and primary and secondary metabolites), we conducted a series of regression analyses. As the percentage of leaf‐damaged areas caused by adult weevils and adult flea beetles were strongly positively correlated with their corresponding densities in the herbivore performance experiments and plant trait analyses (Appendix S1: Figure S2a–d), we therefore used the percentage of leaf‐damaged area instead of herbivore density as the explanatory variable. The response variable of larval flea beetle survival (binomial data) was analyzed using a generalized linear model (GLM) with a binominal distribution and a logit link function, and the response variables of plant root traits (continuous data) were analyzed using linear models (LMs). Because the effects of leaf damage on plant‐mediated herbivore interactions could be nonlinear (e.g., U‐ or hump‐shaped), we first fit two models for each response variable, one with the percentage of leaf damaged area entered as a linear term only and the other with both linear and quadratic damage terms. Then, we selected the model with the best fit based on the lowest Akaike information criterion (AIC; Burnham & Anderson, 2002). Significant effects of the explanatory variables were assessed with *z* tests in GLMs and *t* tests in LMs. Goodness of fit of models were reported as [1 – (residual deviance/null deviance)] in GLMs (Zuur et al., 2009) and the coefficient of determination was reported as *R* ^2^ in LMs. The plant root data were log_*e*_‐transformed where necessary to improve the fit of model residuals. Adult weevil and adult flea beetle herbivory treatments were analyzed separately.

Because a significant quadratic term is not a rigorous test of a U‐ or hump‐shaped relationship (Simonsohn, 2018), we thus conducted additional two‐line tests for models with a significant quadratic term to validate the existence of such relationships (Harman et al., 2020). In this method, we estimated two regression lines, which were interrupted at the break point estimated using the Robin Hood algorithm. U‐ or hump‐shaped relationships could be determined if both regression lines have significant slopes but opposite signs. Larval survival was analyzed based on a binominal distribution with a logit link function. The plant root traits were analyzed based on a Gaussian distribution and transformed as above in their quadratic models.

To investigate whether changes in root quality and quantity might be the causal mechanism underlying plant‐mediated herbivore interactions, we conducted simple linear regression analyses across all feeding treatments to test the dependence of larval flea beetle survival on root biomass, protein, glucose, fructose, starch, or tannin. It should be noted that although plant induced responses are specific to species of attacking herbivore, performance of responding herbivores is mainly dependent on the changes in plant traits (e.g., primary and secondary metabolites). Thus, data from adult weevil and adult flea beetle herbivory treatments were analyzed together.

All analyses were performed in R (version 3.6.3, R Development Core Team, 2020). The package *STATS* was used to fit the GLMs and LMs. For the two‐line test, we used the online R code provided by Simonsohn (2018).

## RESULTS

### Species‐specific impacts of density on herbivore interactions

**FIGURE 1:**
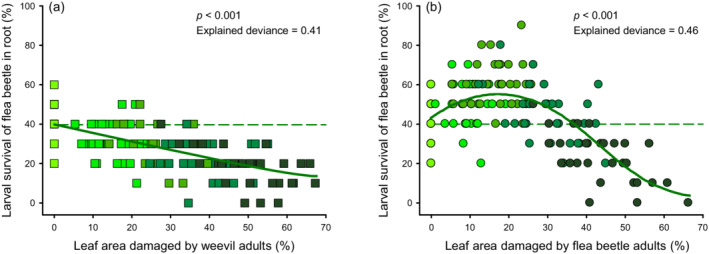
Density‐dependent effects mediate species‐specific induced resistance and susceptibility differently. Relationships between larval flea beetle survival (*Bikasha collaris*) and the percentage of leaf damaged area caused by (a) adult weevils (*Heterapoderopsis bicallosicollis*, squares) or (b) adult flea beetles (*B. collaris*, circles). The best fits included a linear term for the percentage of leaf damaged area of adult weevil herbivory (a), and linear and quadratic terms for the percentage of leaf damaged area of adult flea beetle herbivory (b). Percentage of leaf damaged area = 0 indicates healthy plants. The *p* values and explained deviances are given. Data points represent individual replicates (*n* = 30). Colors from light to dark in green indicate increasing herbivore density (adult weevil density: 0, 2, 4, 6, or 8 per plant; adult flea beetle density: 0, 4, 10, 20 or 32 per plant). The dotted lines indicate larval flea beetle survival on the healthy plants

The best model fit for adult weevil herbivory and larval flea beetle survival was a linear regression (Appendix S1: Table S3). Adult weevil leaf damage decreased larval flea beetle survival (Figure 1a). However, the best model fit for adult flea beetle herbivory and larval flea beetle survival included both linear and quadratic terms (Appendix S1: Table S3). At low damage levels, adult flea beetle herbivory increased larval survival, with the highest survival around 18% of leaf damaged area. However, this positive effect weakened with increasing leaf damage, and beyond around 38%, adult flea beetle herbivory decreased larval survival (Figure 1b). The two lines analysis further showed clear evidence that there was a positive relationship between adult flea beetle herbivory and larval survival at low damage levels (averaged leaf damaged area <18%: slope = 4.89, *p* < 0.001) and a negative relationship at high damage levels (averaged leaf damaged area >18%: slope = −5.82, *p* < 0.001), validating the presence of the hump‐shaped relationship (Appendix S1: Table S4). Thus, the amount of herbivore damage modulated plant‐mediated effects in a species‐specific manner.

### Species‐specific impacts of density on plant traits

**FIGURE 2:**
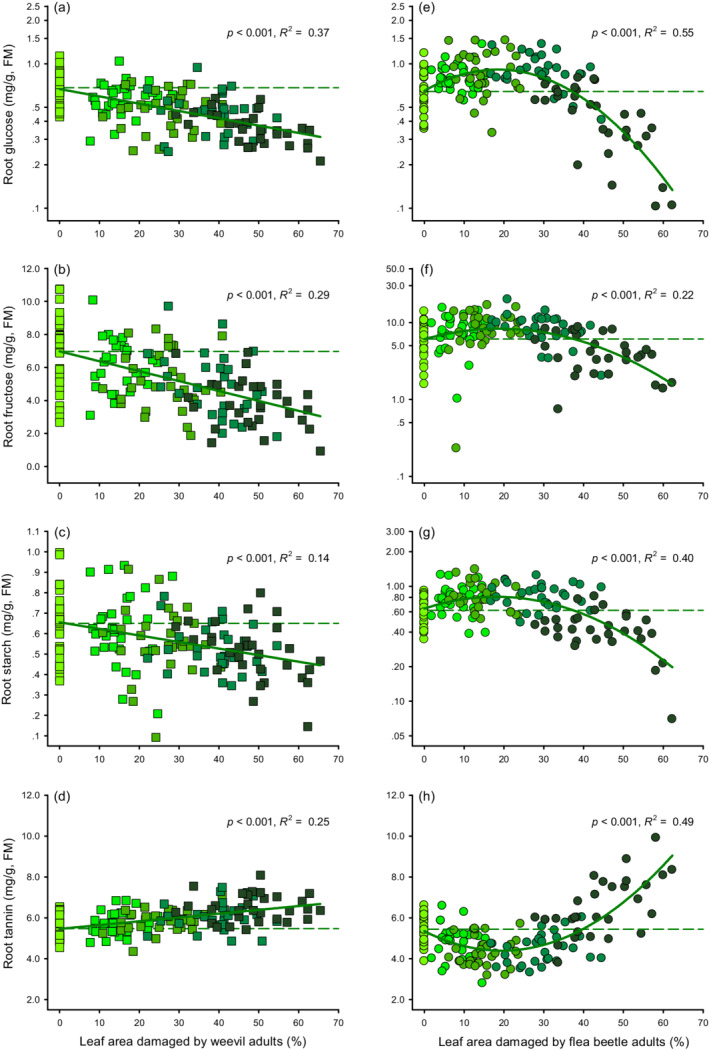
Density‐dependent effects mediate species‐specific plant induced responses differently. Relationships between root glucose, fructose, starch, and tannin and the percentage of leaf damaged area caused by (a–d) adult weevils (*Heterapoderopsis bicallosicollis*, squares) or (e–h) adult flea beetles (*Bikasha collaris*, circles). The best fits included a linear term for the percentage of leaf damaged area of adult weevil herbivory (a–d), and linear and quadratic terms for the percentage of leaf damaged area of adult flea beetle herbivory (e–h). Concentration of glucose (a) in adult weevil herbivory treatment and concentrations of glucose (e), fructose (f), and starch (g) in adult flea beetle herbivory treatments were log_*e*_‐transformed. Percentage of leaf damaged area = 0 indicates healthy plants. The *p* values and *R* ^2^ are given. Data points represent individual replicates (*n* = 30). Colors from light to dark in green indicate increasing herbivore density (adult weevil density: 0, 2, 4, 6, or 8 per plant; adult flea beetle density: 0, 4, 10, 20, or 32 per plant). The dotted lines indicate the concentrations of plant metabolites of the healthy plants. Note the log_*e*_ scale of metabolites (a, e, f, and g) on the *y*‐axis. FM, fresh mass

Consistent with their impact on larval flea beetle survival, the severity of leaf attack by adult weevils had a linear effect on most root metabolites (Appendix S1: Table S5). Concentrations of all root carbohydrates (glucose, fructose, and starch) significantly decreased with increasing leaf damage (Figure 2a–c). Conversely, root tannin increased linearly with increasing leaf damage (Figure 2d). For plants attacked by adult flea beetles, unimodal patterns were observed on most root metabolites (Appendix S1: Table S5), with higher root carbohydrates and lower tannin at low damage levels, and lower root carbohydrates and higher tannin at high damage levels (Figure 2e–h). The two‐line analysis further validated the presence of the hump‐shaped relationship for root carbohydrates and U‐shaped relationship for tannin (Appendix S1: Table S4). Root protein and root biomass were not significantly affected by adult weevil or adult flea beetle attack (Appendix S1: Figure S3).

**FIGURE 3:**
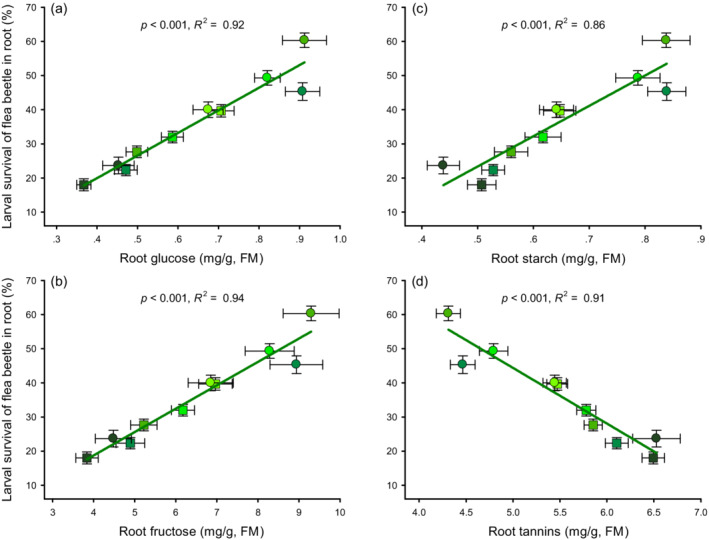
Herbivore survival is strongly associated with root primary and secondary metabolites. Relationships between larval flea beetle survival (*Bikasha collaris*) and tallow root (a) glucose, (b) fructose, (c) starch, and (d) tannin induced by adult weevil (*Heterapoderopsis bicallosicollis*, squares) or adult flea beetle (*B. collaris*, circles) herbivory within an herbivore × density treatment. Values are means ± SE. The *p* values and *R* ^2^ are given. Colors from light to dark indicate increasing herbivory density (adult weevil density: 0, 2, 4, 6, or 8 per plant; adult flea beetle density: 0, 4, 10, 20, or 32 per plant). FM, fresh mass

In addition, larval flea beetle survival for an experimental treatment increased with root glucose (Figure 3a), fructose (Figure 3b), and starch (Figure 3c) concentrations of the treatment but it decreased with root tannin (Figure 3d). Larval survival of an experimental treatment did not depend on root protein (*p* = 0.080) or mass (*p* = 0.157). Thus, the strength of herbivore damage modulated systemic changes in plant primary metabolism and defense in a species‐specific manner, and these induction patterns in turn affected herbivore fitness.

## DISCUSSION

Herbivore identity and density are known to be two major drivers of plant‐mediated interactions among herbivores (Erwin et al., 2013; Underwood, 2000), but their combined effects are poorly understood. This study demonstrates the effect of herbivore density (or damage intensity) on fitness of subsequent herbivores depends on initial herbivore identity and is likely mediated by changes in plant primary metabolism and defense. Therefore, these results help us better understand complex plant‐mediated interactions among herbivores in the field.

We found that adult weevil herbivory decreases larval flea beetle survival in a density‐dependent manner, which is consistent with our prediction that high levels of herbivory intensity would consistently increase the initial induced resistance. Similar results have been also found in many studies that examined effects of induced responses to several different levels of initial attacker density or damage on subsequent herbivores (He et al., 2018; Karban & Baldwin, 1997; Simelane, 2006; Wei et al., 2016). In contrast to adult weevil herbivory, we found that herbivory by adult flea beetles facilitates larval survival at lower feeding intensity, an effect that is reversed at higher feeding intensity. Such shifts have also been observed in other studies (Hausmann & Miller, 1989; Meiners et al., 2005; Pettersson et al., 1998). For example, Pineda et al. (2017) found that *Pieris brassicae* caterpillars preferred the wild crucifer *Brassica nigra* infested by *Brevicoryne brassicae* aphids at low or medium densities over uninfested plants, but preferred uninfested plants to those at high infestation density. Likewise, Goodsman et al. (2015) found that attack by *Dendroctonus rufipennis* beetles increased after moderate *Choristoneura biennis* caterpillar infestation but decreased after severe *C. biennis* outbreaks at the landscape scale. By incorporating two types of herbivores that induced distinct responses in the same plant, we demonstrate, to the best of our knowledge for the first time, that plant‐mediated effects on herbivores are species‐specific at low initial herbivore densities, but converge at high herbivore densities, which is likely to be a common phenomenon among herbivores feeding on the same host plant.

As food resources of herbivores, the amount of available resource can influence the performance of herbivores (Robert et al., 2012b; Walker et al., 2008). However, in the current study, root biomass was not strongly affected by increasing adult flea beetle or weevil feeding intensities, which suggested that observed plant‐mediated density‐dependent interactions between herbivores did not reflect lack of food resources, and thus the potential effect of resource overexploitation in this system was excluded. A similar result was also found in the mustard *Brassica nigra* on which feeding by aboveground herbivore *Pieris brassicae* did not affect root biomass but significantly decreased survival and growth of the belowground herbivore *Delia radicum* (Soler et al., 2007).

In addition, plant quality (e.g., primary and secondary metabolites) also plays a critical role in determining the performance of herbivores (Mithöfer & Boland, 2012; Wan et al., 2019). In this study, adult weevil herbivory consistently reduced three carbohydrates and increased tannin with increasing feeding intensity. In contrast, with adult flea beetle feeding, increased the three carbohydrates and reduced tannin only occurred as feeding intensity was initially increasing, before it attenuated and finally reversed as feeding intensity increased. These findings coupled with the fact that flea beetle larval survival was positively correlated with each carbohydrate and negatively correlated with tannin across all feeding treatments suggested that changes in plant quality might underlie observed plant‐mediated density‐dependent interactions between herbivores (He et al., 2021; Robert et al., 2012b; Soler et al., 2005).

The observed systemic effects on carbohydrates and tannin are likely the combined result of herbivore‐species‐specific plant responses and increasing leaf damage. At low damage, adult weevil and flea beetle infestations caused opposing responses in each of these two types of metabolites, which is consistent with our previous finding about tannin at a comparatively lower intensity of damage (~10%) caused by adult weevils and adult flea beetles separately (Huang et al., 2014). Sarmento et al. (2011) also found similar divergence in proteinase inhibitors, an inducible defense compound, in tomato (*Solanum lycopersicum*) that were separately infested by two spider mite congeners, *Tetranychus evansi* and *Tetranychus urticae* at a single density. Such variation in plant metabolites induced by different herbivore species belonging to the same feeding guild is likely the result of herbivore‐specific cues (Basu et al., 2018; Schuman & Baldwin, 2016). For example, in maize *Zea mays*, attack by the cotton leafworm *Spodoptera littoralis* induced the emission of volatiles while the fall armyworm *Spodoptera frugiperda* suppressed such emission, and similar impacts were further confirmed when their oral secretions were released on artificial damaged leaves (De Lange et al., 2020). We thus hypothesize that adult flea beetles produce specific cues that boost root carbohydrates and suppress tannin, while adult weevils produce cues that have the opposite effect. More work is required to understand the identities and contributions of herbivore‐specific cues of flea beetle adults and weevil adults to damage‐induced responses in tallow.

Interestingly, systemic plant responses in terms of carbohydrates and tannin of the two herbivores converged at high feeding intensities, suggesting that consistently intensified initial herbivore‐induced plant response under high feeding intensity might be only applicable to herbivores that trigger initial induced resistance but not to herbivores that trigger induced susceptibility. In fact, in addition to herbivore‐species‐specific plant responses, damage itself could also induce changes in plant primary and secondary metabolism. On the one hand, removal of plant tissues will decrease photosynthetic activity and nutrient uptake, which, in turn, decreases the plant's nutritional status (Nabity et al., 2009; Zangerl et al., 2002). On the other hand, plants can perceive the elicitors from their own damaged cells and activate general defensive responses (Erb & Reymond, 2019). Both of them have been well demonstrated in many studies using mechanical damage (Erb et al., 2012b; Machado et al., 2017; Mithöfer et al., 2005). For example, in wild‐type tobacco, simulated herbivore feeding by pattern wheels on leaves significantly decreased contents of sugar and starch, but increased content of nicotine in leaves (Machado et al., 2013). Contrary to the induced defenses elicited by herbivore‐species‐specific cues, plant‐induced responses triggered by damage are often assumed to be difficult for herbivores to overcome (Duran‐Flores & Heil, 2014, 2016; Heil, 2009, 2012), and its intensity increases with the amount of damage (Canham et al., 1999; Heil et al., 2001, 2012). Hence, the observed induced responses in plant metabolism and plant‐mediated interactions between herbivores at high damage are probably attributed to the joint effect of increasing herbivore‐induced responses and increasing damage‐induced nutrient limitation and defensive responses.

**FIGURE 4:**
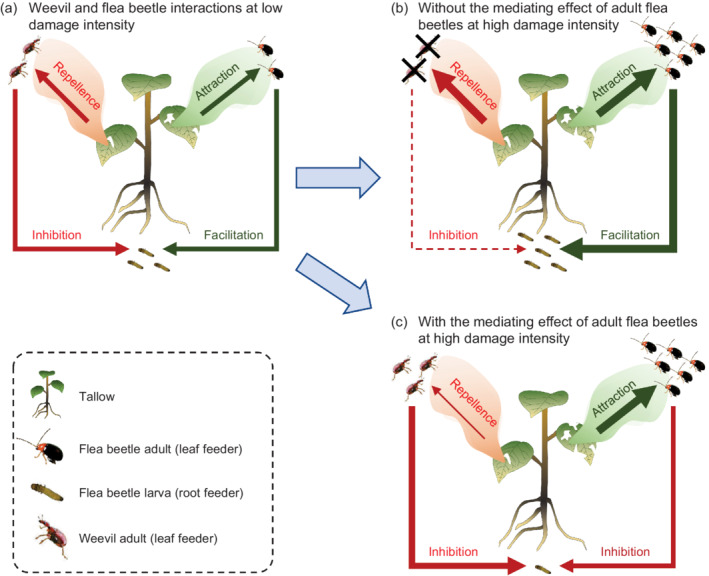
Conceptual models depicting how species‐specific induced responses and ecological consequences depend on damage intensity in system of tallow (*Triadica sebifera*) that is attacked by aboveground flea beetle adults (*Bikasha collaris*) and weevil adults (*Heterapoderopsis bicallosicollis*) as well by belowground flea beetle larvae. (a) At low damage intensities of aboveground herbivores, flea beetle adults and larvae reciprocally facilitate each other's performances via increasing attractive volatiles and root quality (Huang et al., 2012b; Sun et al., 2019), while weevil adults and flea beetle larvae inhibit each other via increasing repellent volatiles and decreasing root quality (Huang et al., 2014; Li et al., 2016; Sun et al., 2019). Mediating effect indicates that the impact of adult flea beetles on their larvae shifts from positive to negative with increasing damage intensity, as shown in this study. (b) Without the mediating effect of adult flea beetles at high damage intensity, increased densities of adult flea beetles would continually promote conspecific larval survival through increasing root quality, which in turn would intensify the negative effects on adult weevils via increased repellent volatiles and finally might exclude weevils from the tallow system. (c) With the mediating effect of adult flea beetles at high damage intensity, increased adult flea beetles would inhibit their larval survival through decreasing root quality, which in turn would weaken negative effects on adult weevils via decreasing repellent volatiles and finally might maintain weevil on the tallow system. Red lines indicate repellence or inhibition; green lines indicate attraction or facilitation

The joint effect of herbivore identity and damage intensity on plant induced responses may be an important mechanism for the maintenance of a diversity of conspecific and heterospecific herbivores in above‐ and belowground compartments (Figure 4). In previous studies at low density of flea beetle larvae, we found that larval flea beetle herbivory induced leaf volatiles attractive to conspecific adults and increased their feeding, while induced leaf volatiles repellent to heterospecific leaf‐feeding herbivores, including adult weevils, which in turn decreased their occurrence, damage, and performance (Figure 4a; Huang et al., 2012b, Huang et al., 2014, Li et al., 2016, Sun et al., 2019). In such conditions, without the mediating effects of adult flea beetles at high density we observed in this study, increased adult flea beetle damage at higher densities would continually increase food quality for larval flea beetles and then promote their survival, which, in turn, would intensify negative effects on adult weevils via increased repellent volatiles. As a result, heterospecific aboveground herbivores might be excluded from the tallow system by the flea beetle and herbivore diversity on tallow would decrease (Figure 4b). However, because of mediating effects of adult flea beetles at high density, increased adult flea beetles can inhibit larval flea beetle survival, which, in turn, might weaken the negative impact on adult weevils through decreasing repellent volatile production, and allow adult weevils to persist on tallow (Figure 4c). Thus, such mediating effects of damage intensity shifting from induced susceptibility to induced resistance for conspecific belowground herbivores may be very important for heterospecific aboveground herbivores, in particular for specialists, such as weevils in this study, because they cannot survive on other host plants (Wang et al., 2009). Future chemical analyses and manipulative field studies would be needed to fully understand such patterns.

In summary, this study shows that herbivore identity and density interact to determine systemic plant responses and plant‐mediated interactions between herbivores. While herbivore‐species‐specific effects on plant responses and subsequent herbivores dominate at low densities, they converge at high herbivore densities. This study highlights the importance of considering herbivore identity and density simultaneously when identifying factors influencing induced plant responses to herbivory and plant‐mediated effects. Furthermore, a large body of studies show that induced plant responses are often systemic (Bezemer & van Dam, 2005; Gutbrodt et al., 2011; Kaplan et al., 2008b). Our findings, therefore, are probably able to be applied broadly, including to interactions among spatially separated herbivores (between leaf and root herbivores) and herbivores feeding on same compartment (between leaf herbivores and between root herbivores). Currently, it is well known that herbivore‐induced changes in the plant's traits can affect species richness and abundance of herbivore communities via both bottom‐up and top‐down effects (Ohgushi, 2005; Turlings & Erb, 2018). Understanding the combined effects of herbivore identity and density will help us further understand the mechanisms that shape the organization and diversity of herbivore communities including conspecific and heterospecific herbivores.

## CONFLICT OF INTEREST

The authors declare they have no conflict of interest.

## AUTHOR CONTRIBUTIONS

Jinlong Wan and Wei Huang conceived the project and designed the research; Jinlong Wan, Jiahui Yi, Zhibin Tao, Zhikun Ren, Evans O. Otieno and Baoliang Tian carried out field survey, herbivore performance experiments and chemical analyses; Jinlong Wan, Evan Siemann, Wei Huang, and Matthias Erb analyzed and interpreted the data; Wei Huang, Jinlong Wan, Jianqing Ding, Evan Siemann,ss and Matthias Erb wrote the initial version of the manuscript, and all authors contributed to its revision and gave final approval for publication.

## Supporting information


Appendix S1

## Data Availability

Data (Wan et al., 2021) are available in Dryad at https://doi.org/10.5061/dryad.cvdncjt5h
