# Supplementary material for: Species‐specific plant‐mediated effects between herbivores converge at high damage intensity
Source: Ecology. 2022 Mar 21;103(5):e3647. doi: 10.1002/ecy.3647 (PMC9285418; doi:10.1002/ecy.3647)
Supplement: Supplementary file 1 — Appendix S1 [file ECY-103-0-s001.pdf]

Supporting Information. Jinlong Wan, Jiahui Yi, Zhibin Tao, Zhikun Ren, Evans O. Otieno, Baoliang Tian, Jianqing Ding, Evan Siemann, Matthias Erb, Wei Huang. Species specific plant-mediated effects between herbivores converge at high damage intensity. Ecology

Appendix S1

Field survey

Method

To examine whether there are wide variations in composition and abundance of herbivores across tallows (*Triadica sebifera*) in real conditions, we conducted a field survey in July 2018 in Dawu, Hubei, China (31.58° N, 114.18°E). We randomly selected 100 tallow plants (80-120 cm in height) along farmland roadsides. The plants were separated by at least 50 m. We carefully checked each leaf and recorded the identity and abundance of herbivores on each plant. We collected herbivores that we could not identify in the field, stored them in glass tubes with 95% alcohol and took them back to laboratory for identification. To quantify natural damage levels, we randomly selected 4 branches that were located diagonally on each plant and visually assessed the percentage of leaf damaged area (to the nearest 5%) for the terminal 10 leaves (40 leaves in total for each plant). A leaf was classified as 100% damaged when it was rolled by weevil adult or when caterpillars had consumed all but the petiole. We determined leaf damaged area of each plant by averaging the visual estimates for all 40 leaves. Leaf damage areas were used for parameterization in the following experiments.

Statistical analyses

To investigate the association between herbivore abundance and tallow leaf damage in the field survey, we performed a simple linear regression analysis across 100 surveyed plants for each herbivore. To account for the interactive impacts between herbivores, we incorporated all herbivores in a multiple regression analysis. Model selection was performed using a stepwise regression with backwards removal based on Akaike's Information Criterion (Burnham and Anderson 2002). *Ricania speculum* was excluded from analysis because it is not a leaf chewing herbivore and does not cause leaf damage that can be measured with our methods. All analyses were performed in R, version 3.6.3 (R Development Core Team 2020). The package 'STATS' was used to conduct the simple and multiple regression analyses. The package 'MASS' was used to conduct the stepwise regression analysis.

Results

In the field, tallow plants experienced a wide range of feeding intensity and the percentage of leaf damaged area ranged from 0 to 71.5% (mean ± se, 19.11 ± 1.64 %; Fig. S1a). There were 16 species found on the leaves of tallow, including eight Lepidoptera species, seven Coleoptera species and one Hemiptera species (Table S1). Flea beetle adult (*Bikasha collaris*) and weevil adult (*Heterapoderopsis bicallosicollis*) were two of the most abundant herbivores, in the proportion of 69.8% and 12.0% in total and with 16.4 and 2.8 per plant, respectively (Fig. S1b). In both simple and multiple regression analyses across 100 surveyed plants, the percentage of leaf damaged area was strongly positively correlated with the abundance of these two herbivores (Table S1-S2, Fig. S1c-d), while there were no significant correlations with abundances of other herbivores (Table S1-S2), suggesting that they are major drivers of leaf damage on tallow.

Table S1 Results of simple linear regressions between the percentage of leaf damaged area and herbivore abundance on 100 surveyed tallow (*Triadica sebifera*) using linear model. Each herbivore was analyzed separately. *Ricania speculum* was excluded from analysis because it is not a leaf chewing herbivore and does not cause leaf damage that can be measured with our methods. Coefficient estimates in each model are presented. The statistical significance was estimated by *t* tests. Significant effects ($P < 0.05$) are shown in bold. SE = standard error.

**Order**	**Family**	**Herbivores**	**Estimate**	**SE**	***t***	**df**	***P***	R^2
Coleoptera	Scarabaeidae	*Anomala* sp.	-0.173	0.165	-1.05	98	0.296	0.011
Coleoptera	Cerambycidae	*Anoplophora chinensis*	0.033	0.096	0.35	98	0.731	0.001
Coleoptera	Cerambycidae	*Batocera horsfieldi*	0.028	0.044	0.64	98	0.523	0.004
Coleoptera	Chrysomelidae	*Bikasha collaris* (Flea beetle adult)	0.004	0.001	4.31	98	**< 0.001**	**0.159**
Coleoptera	Attelabidae	*Heterapoderopsis bicallosicollisis* (Weevil adult)	0.019	0.006	3.19	98	**0.002**	**0.094**
Coleoptera	Scarabaeidae	*Holotrichia trichophora*	0.098	0.117	0.84	98	0.403	0.007
Coleoptera	Scarabaeidae	*Maladera* sp.	0.017	0.074	0.22	98	0.823	0.001
Lepidoptera	Geometridae	*Buzura suppressaria*	0.016	0.053	0.31	98	0.757	0.001
Lepidoptera	Psychidae	*Clania variegata*	-0.003	0.018	-0.15	98	0.878	0.000
Lepidoptera	Limacodidae	*Cnidocampa flavescens*	0.007	0.009	0.73	98	0.465	0.005
Lepidoptera	Erebidae	*Euproctis* sp.	0.009	0.165	0.05	98	0.957	0.000
Lepidoptera	Nolidae	*Gadirtha inexacta*	-0.001	0.006	-0.17	98	0.867	0.000
Lepidoptera	Erebidae	*Grammodes geometrica*	-0.017	0.016	-1.07	98	0.288	0.012

Lepidoptera	Tortricidae	*Homona magnanima*	0.097	0.055	1.77	98	0.079	0.031
Lepidoptera	Noctuidae	*Prodenia litura*	0.004	0.015	0.30	98	0.768	0.001

Table S2 Results of multiple linear regressions between the percentage of leaf damaged area and herbivore abundance on 100 surveyed tallow (*Triadica sebifera*) using general linear model. *Ricania speculum* was excluded from analysis because it is not a leaf chewing herbivore and does not cause leaf damage that can be measured with our methods. The coefficients of each variable are presented for the model with lowest Akaike's Information Criterion (AIC). The statistical significance was estimated by *t* tests. Significant effects (*P* < 0.05) are shown in bold. SE = standard error.

**Species**	**Estimate**	**SE**	***t***	***P***
Bikasha collaris (Flea beetle adult)	0.004	0.001	4.09	**< 0.001**
Heterapoderopsis bicallosicollisis (Weevil adult)	0.017	0.006	2.91	**0.005**
Prodenia litura	-0.021	0.014	-1.44	0.154

Fig. S1 Feeding intensity and herbivore composition and abundance on tallow (*Triadica sebifera*) in the field survey. (a) Number of plants in different leaf damaged category (dam=0, 0<dam≤10%, 10<dam≤20%, 20<dam≤30%, 30<dam≤40%, 40<dam≤50%, 50<dam≤60%, 60<dam≤70%, 70%<dam). (b) Number and ratios of major herbivores found on the leaves of tallow. Others represent the herbivores that proportion was less than 2%. Relationships between leaf damaged area and (c) adult weevil (*Heterapoderopsis bicallosicollis*) abundance, and (d) adult flea beetle (*Bikasha collaris*) abundance. Lines indicate significant linear relationships (*P*-values and R^2 are shown). Data were collected from 100 trees (80-120 cm in height) in the central China.

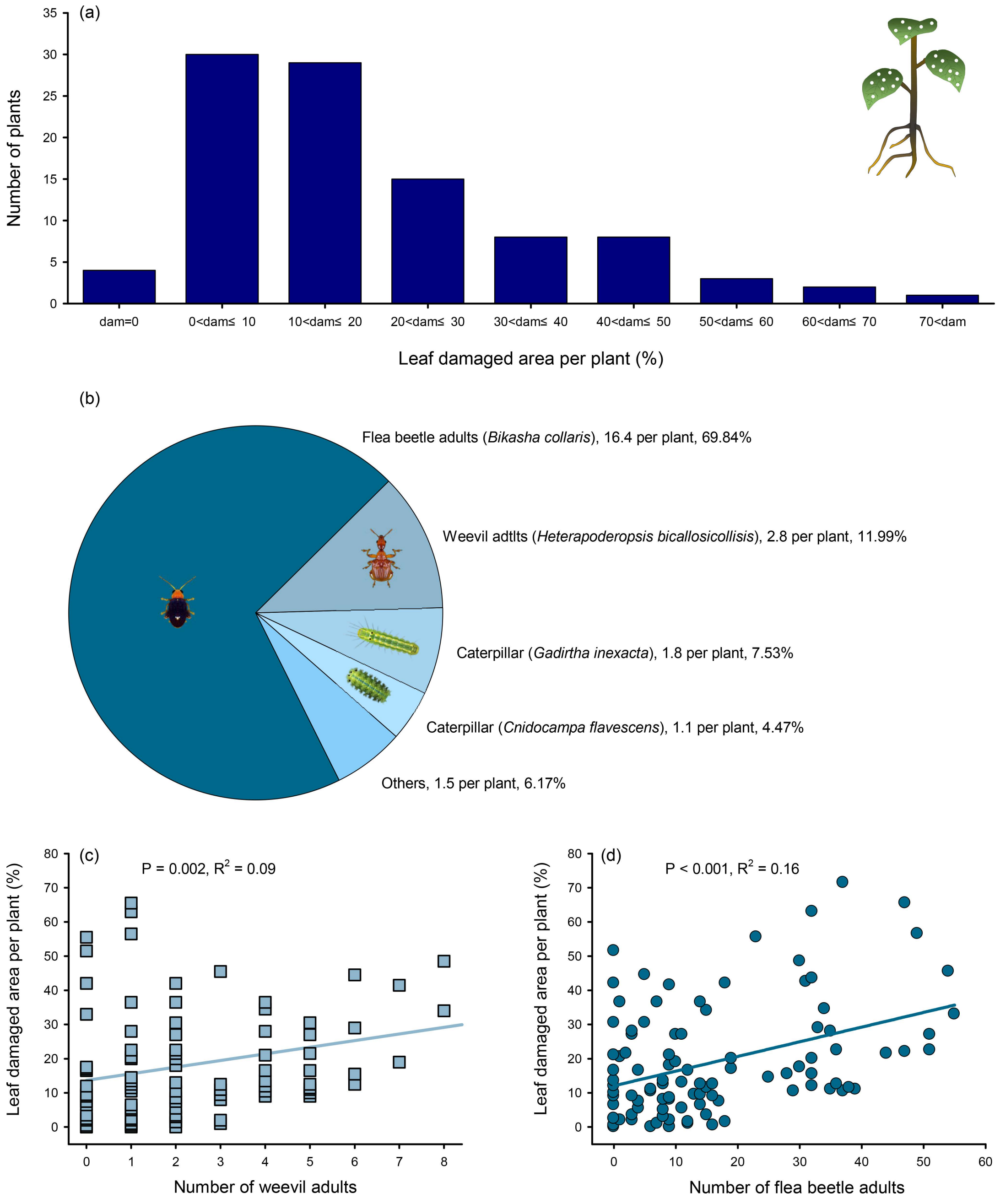

Growth chamber experiments

Table S3 Results of generalized linear models for the percentage of leaf damaged area of tallow (*Triadica sebifera*) caused by weevil adults (*Heterapoderopsis bicallosicollis*) or flea beetle adults (*Bikasha collaris*) on flea beetle larval survival. The percentage of leaf damaged area was entered as a linear or quadratic term. The model with the best fit (in bold) was identified based on the lowest Akaike's Information Criterion (AIC), and its coefficient estimates are presented. The statistical significance was estimated by *z* tests. Explained deviance = 1 - (residual deviance/null deviance). SE = standard error.

**Attacking herbivores**	**AIC for Linear models**	**AIC for Quadratic models**	**Explained deviance**	**Factor**	**Estimate**	**SE**	***z***	***P***
Heterapoderopsis bicallosicollis (Weevil adult)	**443.45**	445.10	0.409	Damage	-2.167	0.318	-6.81	< 0.001
Bikasha collaris (Flea beetle adult)	564.96	**509.10**	0.457	Damage	5.752	1.098	5.24	< 0.001
				$Damage^2$	-16.618	2.369	-7.01	< 0.001

Table S4 Results of the two-lines analyses of larval flea beetle (*Bikasha collaris*) survival and root primary and secondary metabolites of tallow (*Triadica sebifera*) as a function of the percentage of leaf damaged area caused by flea beetle adults. Larval survival was analyzed based on a binominal distribution with a logit link function and root metabolites were analyzed based on a Gaussian distribution. Concentrations of glucose, fructose and starch were $\log_e$-transformed. Break points were estimated using the Robin Hood method introduced by Simonsohn (2018). Slope 1 and 2 represented slopes of regression lines to the left and right of the break point, respectively. U-shaped (or hump-shaped) relationship was determined if *P*-values of both slopes were less than 0.05 and opposite signs. Statistics were carried out using the app available at http://webstimate.org/twolines/.

**Response variables**	**Break point**	**Slope 1**	**Slope 2**	***P*1**	***P*2**	**Damage-dependent pattern**
Survival of flea beetle larvae	0.18	4.89	-5.82	< 0.001	< 0.001	hump-shaped
Glucose	0.23	1.51	-4.82	0.001	< 0.001	hump-shaped
Fructose	0.22	2.59	-3.77	< 0.001	< 0.001	hump-shaped
Starch	0.15	2.01	-2.45	0.002	< 0.001	hump-shaped
Tannin	0.20	-7.24	10.25	< 0.001	< 0.001	U-shaped

Table S5 Results of general linear models for the percentage of leaf damaged area caused by weevil adults (*Heterapoderopsis bicallosicollis*) or flea beetle adults (*Bikasha collaris*) on root primary and secondary metabolites as well as root biomass of tallow (*Triadica sebifera*). The percentage of leaf damaged area was entered as a linear or quadratic term. For adult weevil herbivory treatment, glucose concentration was $\log_e$-transformed; for adult flea beetle herbivory treatment, the concentrations of glucose, fructose and starch were $\log_e$-transformed. The model with the best fit (in bold) was identified based on the lowest Akaike's Information Criterion (AIC), and its coefficient estimates are presented. The statistical significance was estimated by *t* tests. SE = standard error.

**Attacking herbivores**	**Response variables**	**AIC for Linear models**	**AIC for Quadratic models**	R^2	**Factor**	**Estimate**	**SE**	***t***	***P***
Heterapoderopsis bicallosicollis (Weevil adult)	Glucose	**51.02**	52.30	0.369	Damage	-1.166	0.125	-9.31	< 0.001
	Fructose	**600.32**	601.91	0.286	Damage	-6.008	0.781	-7.69	< 0.001
	Starch	**-143.54**	-142.01	0.140	Damage	-0.321	0.065	-4.91	< 0.001
	Protein	**574.07**	575.86	0.001	Damage	-0.309	0.716	-0.43	0.667
	Tannin	**275.23**	277.17	0.251	Damage	1.861	0.264	7.04	< 0.001
	Biomass	**342.13**	344.07	0.004	Damage	0.256	0.330	0.77	0.440
Bikasha collaris (Flea beetle adult)	Glucose	174.88	**90.54**	0.550	Damage	3.691	0.489	7.55	< 0.001
					Damage^2	-10.003	0.935	-10.70	< 0.001
	Fructose	290.11	**266.48**	0.218	Damage	3.321	0.879	3.78	< 0.001
					Damage^2	-8.800	1.681	-5.23	< 0.001

Starch	138.92	**89.50**	0.402	Damage	2.611	0.487	5.36	< 0.001
				$Damage^2$	-7.226	0.932	-7.75	< 0.001
Protein	**521.45**	523.07	0.003	Damage	-0.419	0.660	-0.64	0.526
Tannin	463.72	**386.81**	0.494	Damage	-9.833	1.312	-7.49	< 0.001
				$Damage^2$	25.330	2.511	10.09	< 0.001
Biomass	**350.92**	352.84	0.005	Damage	0.324	0.374	0.87	0.388

Fig. S2 There was a strongly positive relationship between herbivore density and leaf damaged area of tallow (*Triadica sebifera*) in each control experiment. Relationships between density of weevil adult (*Heterapoderopsis bicallosicollis*, squares) and percentage of leaf damaged area that were obtained from herbivore performance experiment (a) and analyses of plant traits (b), respectively. Relationships between density of flea beetle adult (*Bikasha collaris*, circles) and percentage of leaf damaged area that were obtained from herbivore performance experiment (c) and analyses of plant traits (d), respectively. Percentages of leaf damaged area were $\log_e$+1-transformed. *P*-values and R^2 are given. Data points represent individual replicates (n = 30). Colors from light to dark in green indicate increasing herbivore density (weevil adult density: 0, 2, 4, 6, or 8 per plant; flea beetle adult density: 0, 4, 10, 20 or 32 per plant). Note the $\log_e$+1 scale on the y-axes.

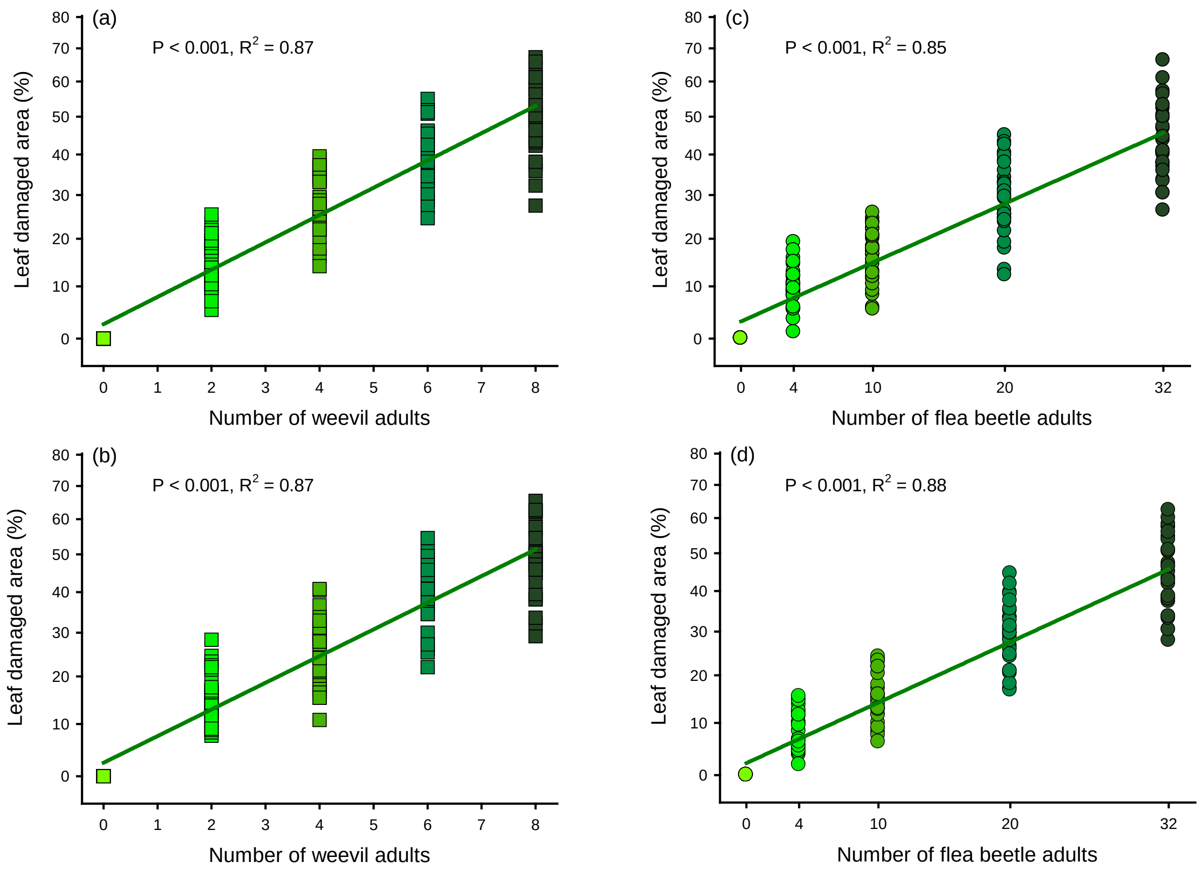

Fig. S3 Density dependent effect does not affect root protein and biomass of tallow (*Triadica sebifera*). Relationships between root protein and biomass and the percentage of leaf damaged area caused by weevil adults (*Heterapoderopsis bicallosicollis*, squares, a-b) or flea beetle adults (*Bikasha collaris*, circles, c-d). Percentage of leaf damaged area = 0 indicates healthy plants. *P*-values are given. Data points represent individual replicates (n = 30). Colors from light to dark in green indicate increasing herbivore density (weevil density: 0, 2, 4, 6, or 8 per plant; flea beetle adult density: 0, 4, 10, 20 or 32 per plant). Dotted lines in (a and c) indicate the concentrations of root protein of the healthy plants and dotted lines in (b and d) indicate root biomass of the healthy plants. FW, fresh weight.

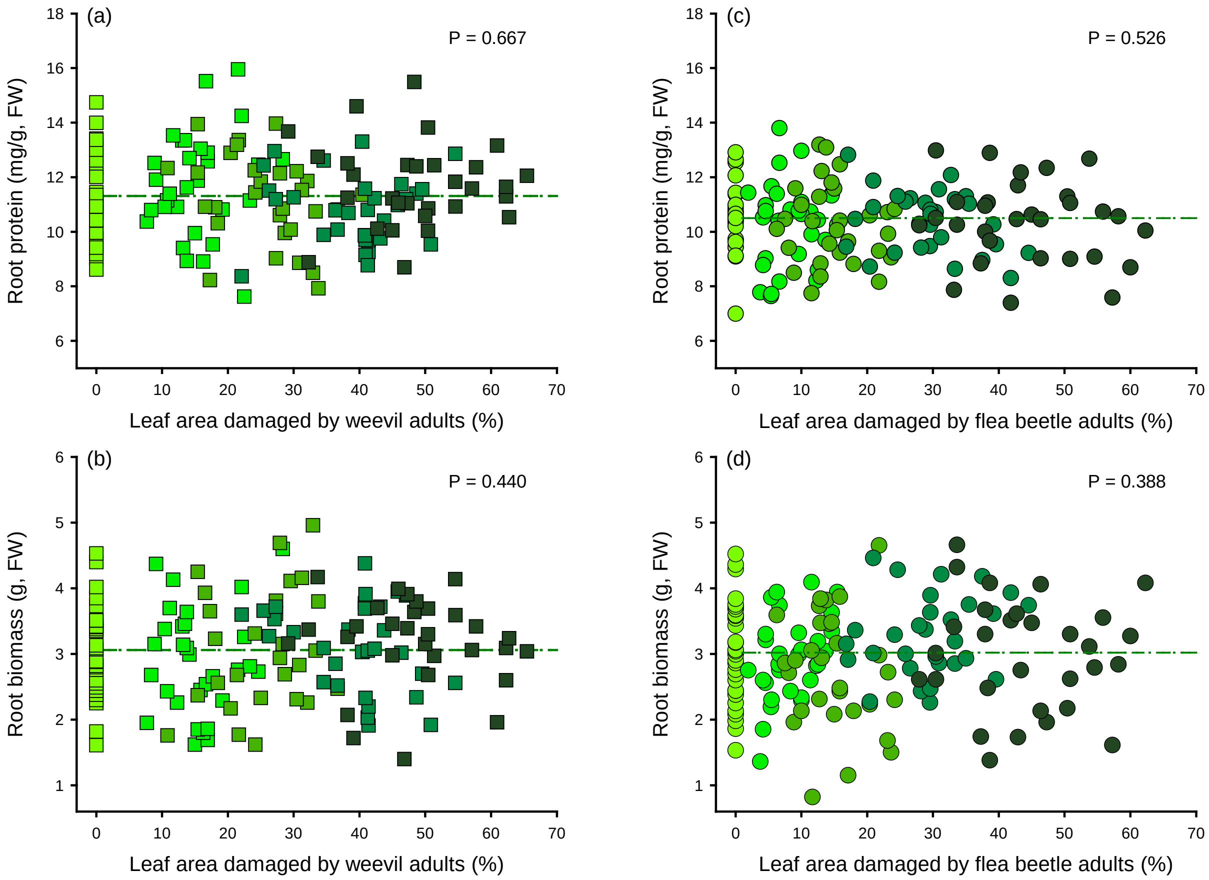

Literature Cited

Burnham, K. P., and D. R. Anderson. 2002. Model selection and multimodel inference: a practical information-theoretic approach. 2 edition. Springer-Verlag, New York, USA.

R Development Core Team. 2020. R: A language and environment for statistical computing. R Foundation for Statistical Computing, Vienna, Austria.

Simonsohn, U. 2018. Two lines: A valid alternative to the invalid testing of U-shaped relationships with quadratic regressions. Advances in Methods and Practices in Psychological Science **1**:538-555.
